# Diagnostic Accuracy of Imaging Findings in Pleural Empyema: Systematic Review and Meta-Analysis

**DOI:** 10.3390/jimaging8010003

**Published:** 2021-12-28

**Authors:** Desiree Zettinig, Tugba Akinci D’Antonoli, Adrian Wilder-Smith, Jens Bremerich, Jan A. Roth, Raphael Sexauer

**Affiliations:** 1 Division of Research and Analytical Services, Department of Informatics, University Hospital Basel, 4031 Basel, Switzerland; desiree.zettinig@ksa.ch (D.Z.); tugba.akincidantonoli@usb.ch (T.A.D.); adrianjonathan.wilder-smith@usb.ch (A.W.-S.); janadam.roth@usb.ch (J.A.R.); 2 Institute of Radiology, Kantonsspital Aarau, 5001 Aarau, Switzerland; 3 Department of Radiology, University Children’s Hospital Basel, 4056 Basel, Switzerland; 4 Department of Radiology and Nuclear Medicine, University Hospital Basel, 4031 Basel, Switzerland; jens.bremerich@usb.ch; 5 Basel Institute for Clinical Epidemiology and Biostatistics, University Hospital Basel, 4056 Basel, Switzerland

**Keywords:** empyema, computed tomography, structured reporting, meta-analysis, pleural findings

## Abstract

Computed tomography (CT) diagnosis of empyema is challenging because current literature features multiple overlapping pleural findings. We aimed to identify informative findings for structured reporting. The screening according to inclusion criteria (P: Pleural empyema, I: CT C: culture/gram-stain/pathology/pus, O: Diagnostic accuracy measures), data extraction, and risk of bias assessment of studies published between 01-1980 and 10-2021 on Pubmed, Embase, and Web of Science (WOS) were performed independently by two reviewers. CT findings with pooled diagnostic odds ratios (DOR) with 95% confidence intervals, not including 1, were considered as informative. Summary estimates of diagnostic accuracy for CT findings were calculated by using a bivariate random-effects model and heterogeneity sources were evaluated. Ten studies with a total of 252 patients with and 846 without empyema were included. From 119 overlapping descriptors, five informative CT findings were identified: Pleural enhancement, thickening, loculation, fat thickening, and fat stranding with an AUC of 0.80 (hierarchical summary receiver operating characteristic, HSROC). Potential sources of heterogeneity were different thresholds, empyema prevalence, and study year.

## 1. Introduction

Pleural effusion is common with an incidence of 0.32% per year in the general population [1] amounting to approximately 1.5 million people in the United States each year alone [2]. Frequently pleural effusion is related to pneumonia, malignancy, or trauma, which may become secondarily infected. Empyema is defined by pus in the pleural space and the most common cause is pneumonia [3]. Empyema-related hospitalizations are increasing [4]. Although empyema accounts for only 5–10% of parapneumonic effusions [5,6], it is associated with worse outcomes: Longer hospital stays and more complications, especially in culture-positive empyemas [7]. Whilst uncomplicated parapneumonic effusions can be treated with antimicrobial therapy, empyema often requires invasive procedures in addition to broad-spectrum antimicrobial therapy [8]. Computed tomography (CT) is a valuable imaging modality for diagnosing pleural effusions and identifying their etiology [9]. Therefore, it is an integral part of diagnostic procedures for a timely diagnosis of empyema.

So far, no systematic review (Cochrane Library, PROSPERO, and PubMed) has evaluated the accuracy of CT for detection of empyema. Therefore, this systematic review and meta-analysis aims to identify relevant CT findings for the diagnosis of empyema and to investigate their diagnostic accuracy including the sensitivity, specificity, diagnostic odds ratio (DOR), and area under the curve (AUC).

## 2. Materials and Methods

This study is registered on PROSPERO (protocol number: CRD42021251903, approved on 29 April 2021). No protocol deviations occurred.

### 2.1. Eligibility Criteria

Based on the PICOT framework, we defined the following inclusion criteria.

- Population: Human patients with empyema as a positive condition and other pleural effusions as a negative condition.
- Index test: Computed tomography.
- Comparison: Diagnosis based on positive culture or gram-stain, pathological, or macroscopic confirmation [10,11,12].
- Outcome: Diagnostic accuracy measures (e.g., sensitivity, specificity, area under the curve (AUC), diagnostic odds ratio (DOR)). The data is retrievable to calculate a 2 × 2 contingency.
- Time-period: Studies between 01-1980 and 10-2021.

Case reports, case series, and animal experiments were excluded.

### 2.2. Information Sources

Information sources were Pubmed, Embase, and Web of Science (WOS).

### 2.3. Search Strategy

A sensitive search strategy was established with Mesh-term and Title/Abstract search in Pubmed which included the terms “empyema”, “computed tomography”, and “diagnostic accuracy”. This search strategy was translated with the “polyglot search translator” [13] to “Embase” and “Web of Science”. The detailed search terms can be found in Appendix A. The literature search was updated monthly, with the last update performed on 31 October 2021. Additionally, “Cochrane library”, PROSPERO, and online clinical trial registries such as ClinicalTrials.gov (https://clinicaltrials.gov, last update: 31 October 2021) and ISRCTN (https://www.isrctn.com, last update: 31 October 2021) were searched for additional relevant studies.

### 2.4. Selection Process

Eligibility screening was conducted in two steps: Title and abstract screening for matching the inclusion criteria (1) and full-text screening (2)

Title, author, and abstract were exported from Pubmed, Embase, and WOS to Microsoft Excel 2019 (Redmond, WA, USA). Duplicates were removed prior to the initiation of the screening process. Both reviewers independently reviewed the title and abstract of all identified studies blinded to each other.

If disagreement existed or a paper could not be excluded by title and abstract alone, the paper was included for full-text reading. Full-text versions of relevant studies were retrieved for further evaluation. Reference lists of included studies were checked manually to identify other relevant papers.

### 2.5. Data Collection Process

A structured data extraction sheet [14] was designed, which included QUADAS-2 [15] and all STARD 2015 [16] criteria to review the identified studies summarized in Appendix B. Assessment of risk of bias and methodological quality is summarized in Appendix C. A study was judged to be at risk of bias if one or more QUADAS criteria were unclear or high.

### 2.6. Data Items and Data Extraction

Both reviewers assessed both the individual data items and risk of bias in the uniform data extraction sheet in a blinded design. Any disagreement was resolved by rechecking the original data and consensus.

### 2.7. Statistical Analysis and Data Synthesis 

All statistical analyses including synthesis methods were performed with R 4.0.5 (R Core Team, Vienna, Austria) and the following packages: “mada”, “ellipse”, “meta”, “metafor”, “rmeta”, “tidyverse”, and “mvtnorm”.

For each study included in the meta-analysis, data were extracted to generate 2 × 2 contingency tables displaying true positives, true negatives, false positives, and false negatives. Patients without infected pleural effusion were regarded as disease negatives and patients with a positive culture, gram stain, or macroscopic pus as disease positive. False positives were defined as patients having the disease based on a positive pleural finding but categorized as not having the disease by the reference standard.

Pooled sensitivity, specificity, DOR, and AUC (univariate and hierarchical analysis), as well as 95% CI intervals, were calculated for each pleural finding of the published studies. Forest plots were constructed for all included studies displaying sensitivity and specificity.

Since a common implicit cut-off value for test positivity is to be expected and large differences between disease prevalence in different studies exist, estimates of pooled sensitivity and specificity were calculated by fitting a bivariate random effect model to account for both within- and between-study heterogeneity [17,18]. We quantified heterogeneity between the studies using the I^2^-Index and level of heterogeneity (low < 25, moderate 25–75, and high > 75) as defined by Higgins et al. [19]. We are aware there is a threshold value effect for diagnostic accuracy studies of modalities so that these can only be interpreted to a limited extent [20].

Informative CT findings were defined as a DOR 95% confidence interval, not including 1 [21]. The publication bias could only be assessed to a limited extent, as there is no generally accepted method for diagnostic accuracy studies and the number of studies included was low [22]. Subgroup analyses for sensitivity and specificity with random effect models were performed regarding informative pleural findings, the negative collectives (parapneumonic effusions, benign effusions, or effusions in general), concerns regarding applicability (QUADAS-2), the reference standard, slice thickness, whether a study was performed after the year 2000, multiple reviewers, and the dichotomized prevalence of empyema (cutoff 30%). Additionally, a meta-analysis with a mixed-effects model based on DOR estimates was used for disease prevalence and study year. We evaluated suspected significance based on meta-regression with permutation tests (1000 iterations). Alpha level was set to 0.05.

## 3. Results

### 3.1. Study Selection

**Figure 1:**
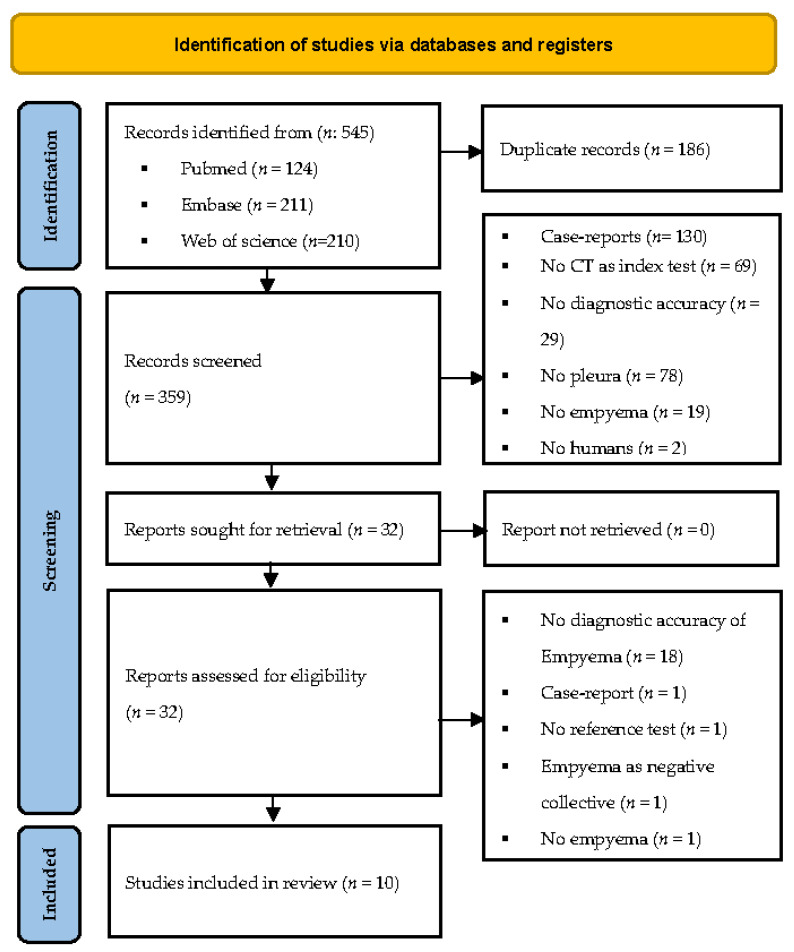
Study flow chart according to PRISMA [45].

The initial search identified 545 studies, which were screened by title and abstract after deduplication. Figure 1 shows the study flow detailing search results and study inclusion. No comparable study was found on Cochrane library, Clinical Trials, or Prospero. A total of 32 articles were eligible for full-text screening and were examined in detail according to the pre-specified PICOT criteria. A manual search of references from these studies and reviews did not yield any additional records. A total of 22 were excluded (see Table A1) after full-text assessment for the following reasons: No diagnostic accuracy design [23,24,25,26,27,28,29,30,31,32,33,34,35,36,37,38,39,40] (*n* = 18), no empyema in the study collective [41] (*n* = 1), case-report [42] (*n* = 1), no reference test [43] (*n* = 1), and empyema as negative collective [44].

### 3.2. Data Extraction/Characteristics of the Included Studies Population

Finally, 10 studies were included in the quantitative synthesis (meta-analysis) with a total of 1098 patients and 252 empyemas. The summary of the baseline characteristics is shown in Table 1. The mean patient age ranged from 56 to 72. All studies were a retrospective cohort study design.

### 3.3. Risk of Bias

The quality of included studies assessed by QUADAS-2 is summarized in Table 2. As illustrated, there is a substantial amount of underreporting in the included studies, resulting in many “unclear” judgments which consequently diminish the quality of the data. None of the studies reported whether the reference standard was blinded for the index test.

### 3.4. Categorization of Pleural Findings

**Figure 2:**
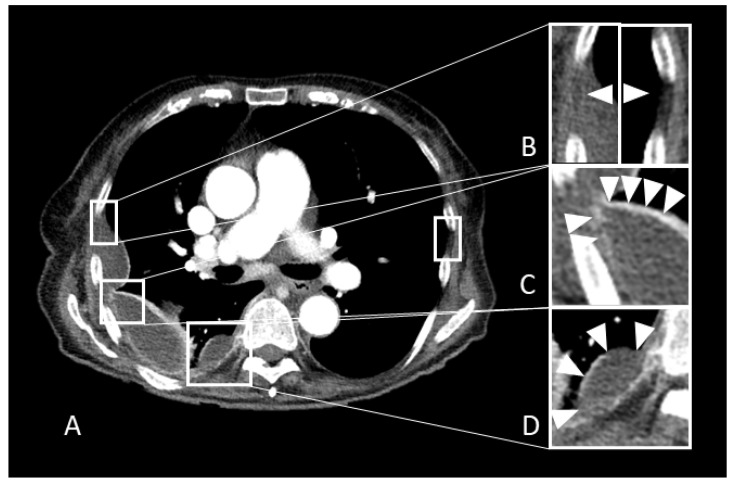
Pathological confirmed pleural empyema of an 83-year-old female patient. (**A**): Original axial slice with empyema on the right side. (**B**–**D**) Magnifications views with (**B**): Pleural fat thickening and increased attenuation (fat stranding) compared to the contralateral side. (**C**): Pleural thickening with an increased enhancement of the pleura. (**D**): Loculation (biconvex, acute marginal angles).

There were 119 overlapping descriptions of which 99 describe the pleura, pleural effusion, or the adjoining adipose tissue, and 20 other findings such as lymphadenopathy, liver metastases, lung metastases, and pneumonia. Of these, duplicates were removed and 35 CT findings were assessed as descriptors of empyema. Of these findings, 11 findings were not included in the meta-analysis because they were described in less than 2 studies with the same negative collective (parapneumonic effusion, benign effusion, or pleural effusion in general). Table A2 summarizes the descriptors that were not used for the meta-analysis. Finally, similar descriptors (*n* = 24) referring to the same imaging finding were subsumed under the following five informative CT findings (visually summarized in Figure 2) after consensus discussion: Pleural enhancement (including the split pleura sign), “pleural thickening” (visible—4 mm), “loculation”, “fat thickening” (visible—4 mm), and “fat stranding”. Sensitivity, specificity, and DOR are summarized in Table A3. “Hemisplit pleura sign”, “circumferential pleural thickening”, “pleural thickening ≥ 4 mm”, and “fat thickening > 5 mm” were identified as non-informative (2.5% DOR ≤ 1) and later excluded from the following analyses.

### 3.5. Results of Syntheses

Sensitivities for informative pleural findings independent of negative collective were 84% (95% CI 62–94) for pleural enhancement, 68% (95% CI 56–77) for pleural thickening, 52% (95% CI 44–59%) for loculation, 53% (95% CI 47–60) for fat thickening, and 39% (95% CI 32–48) for fat stranding, with corresponding specificities of 83% (95% CI 75–89), 87% (95% CI 80–92), 89% (95% CI 82–94), 91% (95% CI 72–96), and 97% (95% CI 94–98), respectively. The “split pleura sign” as a specific threshold for pleural enhancement was explicitly addressed in 2 studies [45,46] with a pooled sensitivity of 68% (95% CI 51–81) and a specificity of 83% (95% CI 71–91).

**Figure A1:**
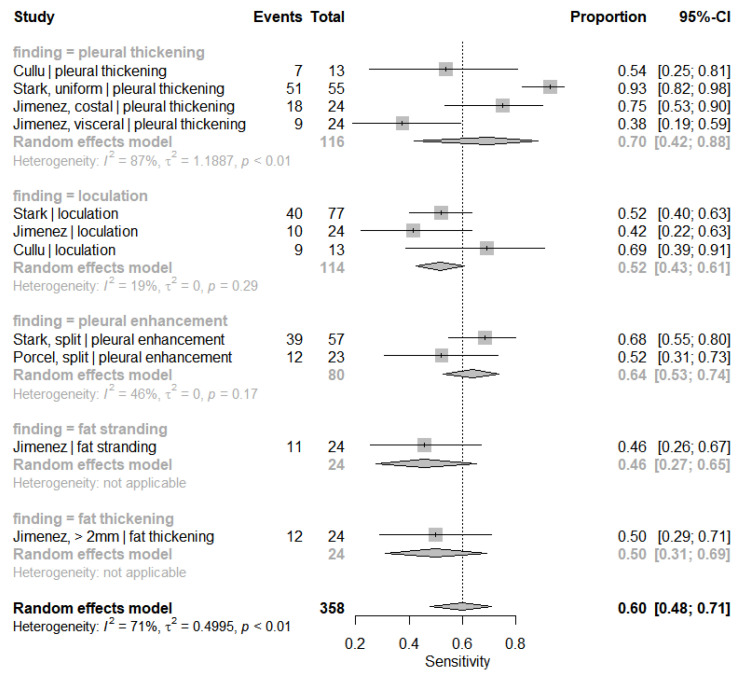
Sensitivity of informative pleural findings to detect pleural empyema in parapneumonic effusions.

**Figure A2:**
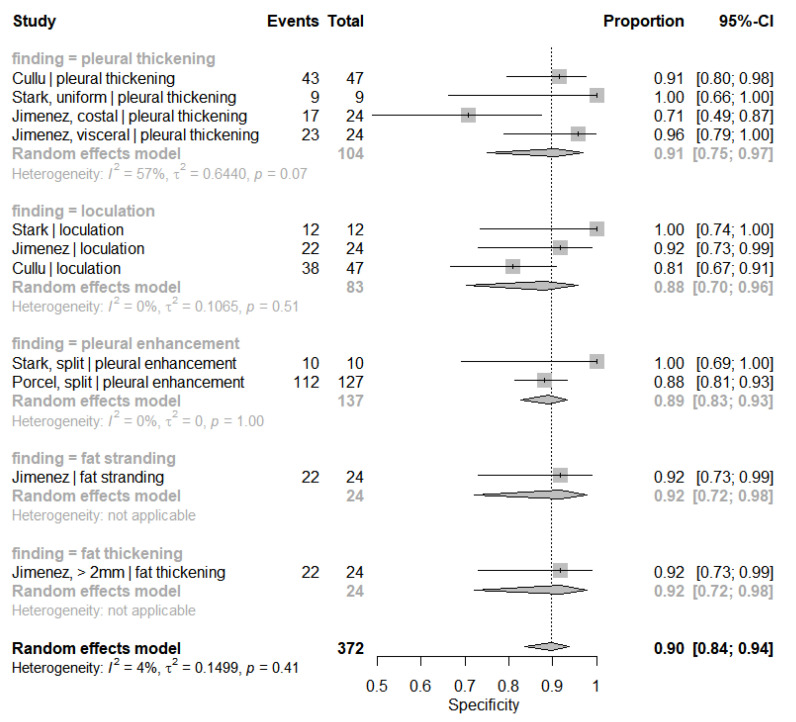
Specificity of informative pleural findings to detect pleural empyema in parapneumonic effusions.

**Figure A3:**
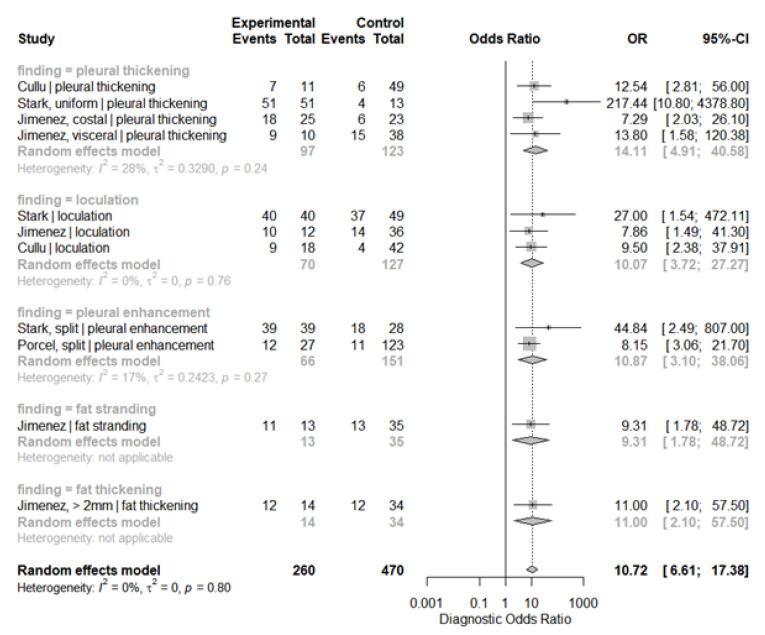
DOR of informative pleural findings to detect pleural empyema in parapneumonic effusions.

**Figure A4:**
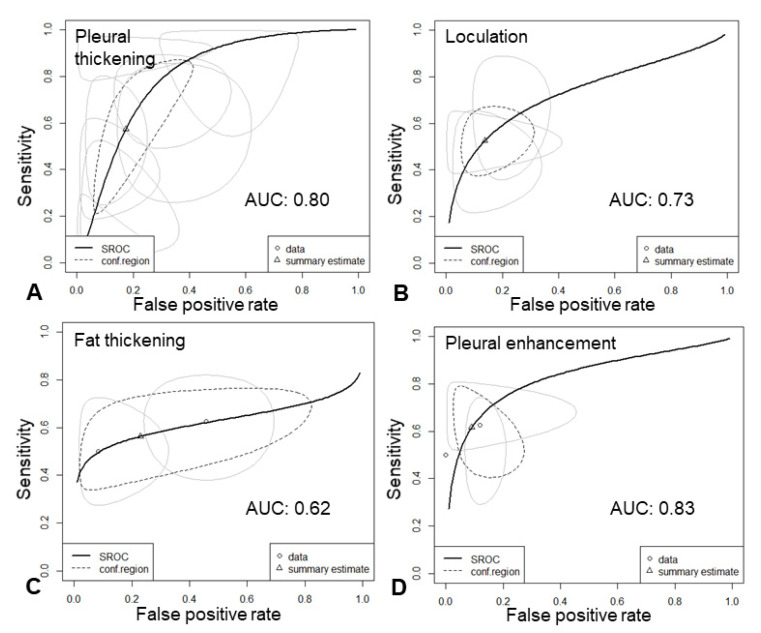
HSROC in parapneumonic effusions: HSROC curve in black with confidence region (dashed line) of the pleural findings (**A**): Pleural thickening, (**B**): Loculation, (**C**): Fat thickening and (**D**): Pleural enhancement (each shown as a point and 95% confidence region as a gray ellipse).

Table 3 summarizes the syntheses of the pleural findings. In addition, we analyzed the diagnostic accuracies of the negative collective for parapneumonic (Figure A1, Figure A2, Figure A3 and Figure A4), benign, and effusions in general (Table A5). For the distinction between empyema and parapneumonic effusion, pleural enhancement and thickening have the highest specificities (89% and 90%) with the highest AUCs (bivariate: 0.83 and 0.80). Figure A6 shows a scatter plot of the studies’ observed sensitivities against their standard error without significant asymmetry (only informative CT findings, Eggers Test: intercept = 0.70, t = 0.28, *p* = 0.786).

### 3.6. Empyema and Subgroup Analysis

**Figure A5:**
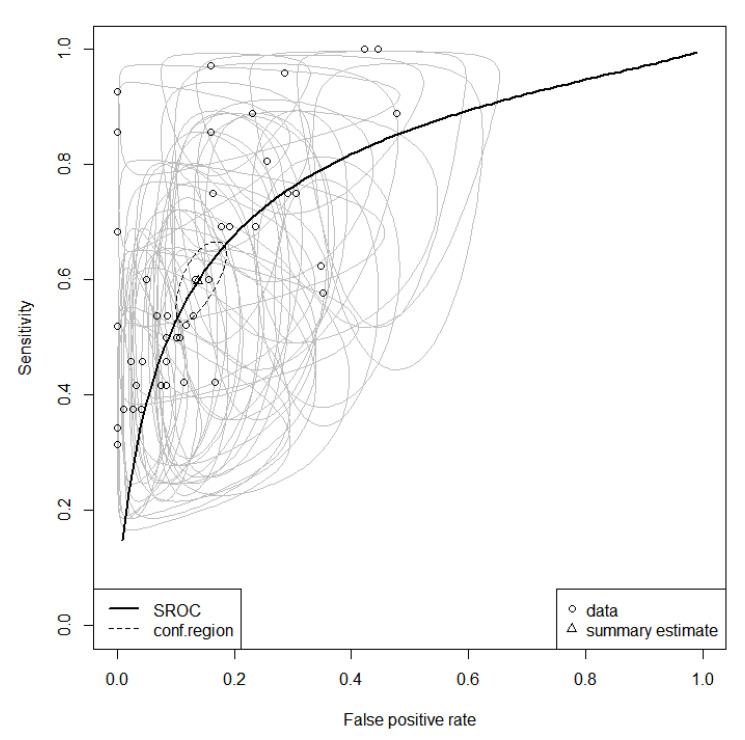
HSROC for all pleural findings and all negative collectives: HSROC curve in black with confidence region (dashed line) of all pleural findings (each shown as a point and 95% confidence region as a gray ellipse) with an AUC of 0.80.

**Figure A6:**
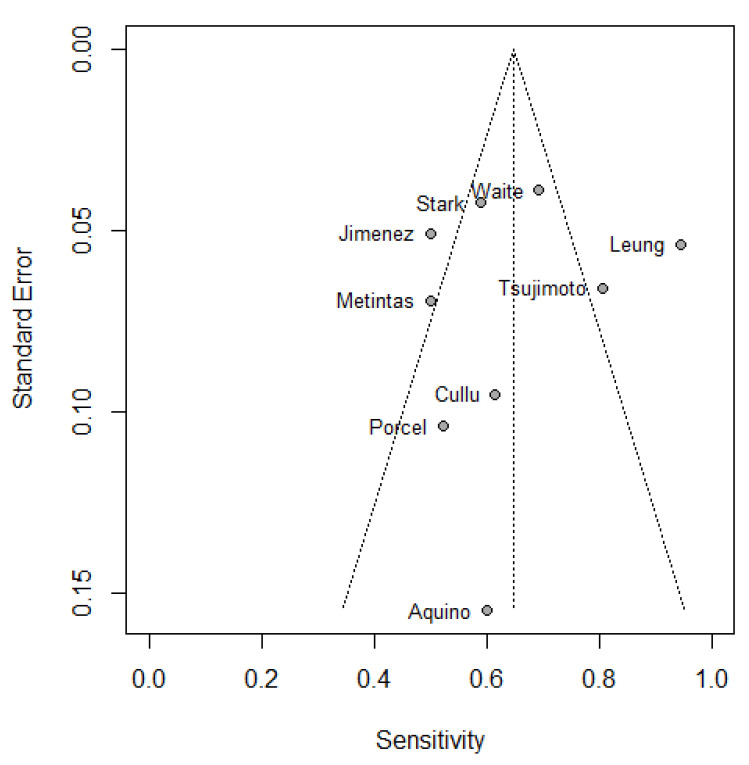
Scatter plot of sensitivities of the studies (only informative CT findings) compared to their standard error. The summary estimate is based on a random effects model.

If the CT findings are interpreted as different threshold values for the same diagnosis of empyema, the result is a pooled specificity of 90% (95% CI 86–93) and a sensitivity of 62% (95% CI 55–68) with an AUC of 0.80. Figure A5 shows the corresponding HSROC curve.

The individual pleural finding (*p* ≤ 0.001 for sensitivity and specificity), the prevalence of empyema (*p* = 0.04 for specificity), slice thickness (*p* < 0.001 for sensitivity), and whether a study published after 2000 (*p* = 0.01 for specificity) was identified as a source of heterogeneity with significant differences in pooled diagnostic accuracy measures of the subgroups.

Based on the random-effects model, there is a significant difference between the sensitivity (*p* ≤ 0.001) of the individual pleural findings, ranging from 84% for pleural enhancement to 39% for fat stranding. There is also a significant difference between the specificity (*p* ≤ 0.001), ranging from 83% for pleural enhancement to 97% for fat stranding. Sensitivities (84%, 68%, *p* = 0.14) and specificities (83%, 87%, *p* = 0.40) of pleural enhancement and pleural thickening do not differ significantly.

The empyema prevalence between the studies ranged from 11% [47] to 87% [48] with a significant effect on specificity (*p* = 0.04), and with a pooled specificity of 94% (95%CI: 88–97%) for studies with a prevalence > 30% versus 87% (95%CI 81–91%) < 30%. Mean prevalence was 34% compared to an expected prevalence of ~10% in parapneumonic effusions [5]. The mixed-effect model was significant for prevalence (0.01, tau^2^: 0.14, sampling variability H^2^: 1.28, residual heterogeneity I^2^: 21.87%), which accounts for 23.75% (R^2^) of heterogeneity.

The following slice thicknesses were used in the studies: 10 mm [49,50,53], 6.5–10 mm [48], 6–10 mm [54], 1.5–10 mm [51], 5 mm [52], and 3 mm [46], with a pooled specificity of 94% (95%CI 87–98%), 92% (95%CI 87–95%), 85% (95%CI 78–90%), 58% (95%CI 50–65%), and 88% (95%CI 81–93%). Sensitivities did not defer significantly (*p*: 0.634) with 66% (95%CI 52–77%) for 10 mm, 62% (95%CI 50–72%) for 5 mm, and 52% (95%CI 32–71%) for 3 mm.

**Figure 3:**
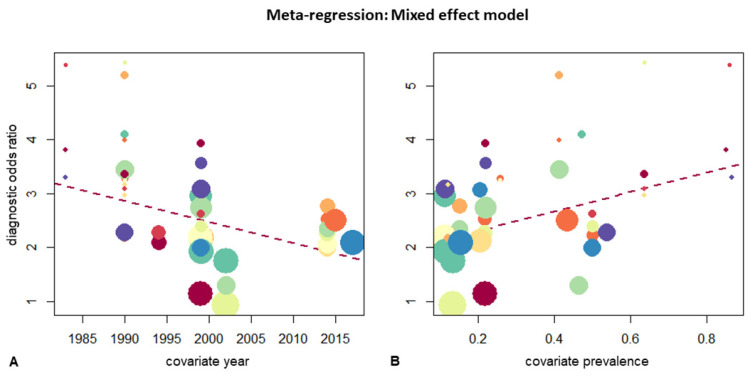
Mixed effect model for the moderator’s (**A**): “year” and (**B**): “empyema prevalence”. The dotted red line shows regression.

Studies after 2000 showed higher pooled specificity with 92% (95%-CI 87–95%) compared to 84% (95%CI 78–88%), with an inverse tendency in sensitivity of 59% (>2000; 95%CI 50–67%) compared to 63% (<2000, 95%CI 54–71). The mixed-effect model (*p* = 0.02) estimated the amount of heterogeneity to be 4.92% (R^2^) for the year of publication (residual heterogeneity I^2^: 25.8%, sampling variability H^2^: 1.35). Figure 3 shows the metaregression for the covariate’s year and prevalence.

There was no significant difference between the negative collectives (sens: *p* = 0.96/spec: *p* = 0.84), the reference standard (sens: *p* = 0.26/spec: *p* = 0.99), and between the number of reviewers (sens: *p* = 0.75/spec: *p* = 0.24). A tabular representation of subgroup analysis can be found in Table A6.

## 4. Discussion

Informative CT findings had visible pleural enhancement (including split pleura sign), pleural thickening (<4 mm), loculation, subcostal fat thickening (<4 mm), and fat stranding. With those findings, detection of empyema using CT has a pooled specificity of 90% (95% CI 86–93), a sensitivity of 62% (95% CI 56–68), and an AUC of 0.80. Of those informative findings, pleural enhancement and pleural thickening had the highest sensitivities with 84% (95% CI 62–94) and 68% (95% CI 56–77), respectively, whereas fat stranding and fat thickening showed the highest specificities of 91% (95% CI 72–96) and 97% (95% CI 94–98), respectively.

Of the subsumed pleural findings, pleural enhancement and fat stranding had the highest DOR with 20.1 and 26.5. Smooth margin, microbubbles, or pleural gas showed relative high DORs in the narrative summary (range: 5.6 [46,48]–62.4 [49,50]). Despite comparable feature-definitions, there were frequently major differences in the DOR. For example, the DOR of visible fat stranding varied between 28.8 [48] and 19.2 [53] and the DOR of the “Split pleura sign” varied between 7.9 [46] and 44.8 [49]. The diagnostic value of the amount of effusion [47,50] and the presence of septations [47,49] remains unclear, as the available studies show controversial results with regard to the DOR.

While different studies used different CT findings to indicate thoracocentesis [46,56,57], the identified informative findings can be used to differentiate empyema from other pleural diseases in a more complete and standardized manner. This distinction is important because both clinical management and patient outcomes differ [10,58]. Because pleural effusions are managed conservatively, false-negative empyema diagnoses should be avoided, suggesting that more value should be given to sensitivity over specificity. Most of the included studies lacked detailed definition and description of CT findings [46,47,48,49,50,51,52], thereby limiting the analysis of different thresholds. However, since CT findings have relatively high specificity with lower sensitivity, no other lower threshold value can be recommended besides the visibility of the findings. However, a threshold greater than 4 mm for pleural thickening [53,54] and subcostal fat thickening [53] was not shown to be informative, mainly as this decreases the differentiability from a pleural tumor manifestation. Whereas pleural carcinomatosis is more likely to show nodular, rind-like, pleural thickening (>10 mm) [50,51] or a pleural-based soft tissue mass [50,51], empyema tends to show smooth pleural thickening [48,54].

In an attempt to maximize pleural enhancement, a dedicated CT protocol is warranted [59,60] to further increase the sensitivity of pleural enhancement and pleural thickening at the expense of a potential higher false-positive rate. In addition, more specific features including fat thickening and fat stranding should be utilized to achieve a higher overall diagnostic accuracy. With newer CT scanners and modern diagnostic monitors offering higher resolution, an ever-increasing higher sensitivity can be expected. Surprisingly, our study showed an inverse correlation when comparing sensitivity with the study date as well as no significant difference with decreasing slice-thickness. This could be partly explained by the fact that older studies only partially fulfilled the STARD criteria, and the patient flow in the included studies remained mostly unclear.

There are several limitations to this study. First, the number of included studies was limited, resulting in a paucity of data available for meta-analysis. Second, different CT parameters, especially concerning the administration of contrast medium, could only be compared to a limited extend, as these were not recorded in a standardized manner in the studies presented. This also applies to the slice thickness, as several studies used different CT scanners or CT settings and therefore only overlapping subgroups could be formed. Finally, we found high heterogeneity among the studies used, which can only be partially explained by the subgroup analyses. This might be mostly related to poor methodology and serious underreporting of the patient selection process. This is an important cause of concern and should be taken into consideration when interpreting the results

## 5. Conclusions

Our study concludes that an early diagnosis depends on a high index of suspicion. Combined with the presence of one (or more) of the several aforementioned informative pleural findings, the diagnosis of pleural empyema can be made with high specificity.

## 6. Future Directions

Imaging advances and a lack of evidence for the optimization of CT protocols with regards to contrast agent administration indicate the need for further studies. In addition to confirming the high specificity already shown in our review, this could lead to improvements in sensitivity. The CT imaging, which is often performed routinely, could thus become increasingly reliable and useful for therapy decisions in the management of pleural empyema.

## Figures and Tables

**Table 1:** Descriptive statistics of the included studies.

	Study								Index Test: CT									Reference Standard
	Journal	Year	Duration	OCEBM	Included (n)	Mean Age (y)	Female (n)	Empyema (n)	Vendor *	i.v. Contrast (n)	Contrast Agent **	Delay (s)	i.v. (mL)	Rate (mL/s)	Slice Thickness (mm)	Rater (n)	Experience (y)	Procedure ***
Porcel [46]	APSR	17	08–15	2	150	56	NA	23	IV	150	b/c	~60	90–100	3	3	2	20 & 20	2 B
Tsujimoto [47]	PloS one	15	06–14	2	83	72	13	36	NA	23	NA	NA	NA	NA	NA	4	10	1/2 B
Jimenez [48]	ER	99	NA	2	211	63	66	24	II/III/VIII	211	c	NA	100–120	2–3	6.5–10	2	NA	2/3 B
Stark [49]	AJR	83	NA	4	63	NA	NA	58	I	NA	a	NA	150	NA	10	3	NA	1 (53%), A
Metintas [50]	EJR	02	89–98	2	215	NA	NA	26	V	215	NA	NA	NA	NA	10	4	NA	2 B/C
Leung [51]	AJR	90	85–89	2	74	60	21	9	I/II	58	NA	NA	NA	NA	10	2	NA	2 B
Cullu [52]	DIR	14	10–12	3	106	NA	46	13	IX	58	f	NA	100–300	2–3.5	1	2	NA	2 B
Waite [53]	Radiology	90	NA	2	85	57	NA	35	I/II	75	a	~20	120	0.9	10	NA	NA	2 B
Aquino [54]	Radiology	94	NA	2	80	58	25	10	II/VI	80	d	NA	60–200	1.7	6–10	2	NA	2 B
Takasugi [55]	BJR	91	NA	2	24	NA	NA	18	VII	14	e	NA	170	NA	10/30	NA	NA	1/2 B/D

Missing data are marked NA. * I: GE 8800 with a 10-mm slice thickness (ST), II: GE 9800 with a 10-mm ST, III: GE Pace Plus with a 6.5–10-mm ST, IV: Philips Brilliance with a 3-mm ST, V: Toshiba TCT 600 with a 10-mm ST, VI: Imatron Cine Scanner with a 6–8-mm ST, VII: Pixer 1200 SX with a 10-mm ST, VIII: Elscint Helicat II with a 6.5–10-mm ST, IX: Siemens Somatom emotion with a 5-mm ST. Note: I-III, V-VIII: Single slice, IV: 16/64 slice, IX: 16 slice. **: a: Diatrizoate meglumine, b: Iobitridol (Xenetix, Guerbet), c: Iopromide (Clarograf, Bayer), d: Iohexol (international nonproprietary name), e: Iothalamate meglumine (Conray, Guerbet), f: Iopamidol (international nonproprietary name). *** 1: Thoracotomy, 2: Thoracocentesis, 3: Biopsy, A: Clinical diagnosis, B: Culture/gram stain, C: Macroscopic purulent pleural fluid, D: Laboratory findings (pleural LDH/WBC/protein). Abbreviations: OCEBM-Level (Oxford Centre for Evidence Based Medicine). Y: Years. I.v.: Intravenous.

**Table 2:** Evaluation according to the QUADAS-2 criteria.

	Risk of Bias				Applicability Concerns		
Study	Patient Selection	Index Test	Reference Standard	Flow and Timing	Patient Selection	Index Test	Reference Standard
Porcel [46]	low	low	unclear	unclear	low	low	low
Tsujimoto [47]	unclear	low	unclear	low	unclear	low	low
Jimenez [48]	low	low	unclear	high	low	low	low
Stark [49]	high	low	unclear	high	unclear	low	high
Metintas [50]	low	low	unclear	high	low	low	low
Leung [51]	low	low	unclear	high	low	low	low
Cullu [52]	unclear	high	unclear	low	unclear	unclear	unclear
Waite [53]	low	unclear	unclear	low	low	low	low
Aquino [54]	low	low	unclear	low	low	low	low
Takasugi [55]	unclear	low	unclear	unclear	unclear	low	low

**Table 3:** Syntheses of the pleural findings with the pooled sensitivities and specificities.

	Enhancement	Pleural Thickening	Loculation	Fat Thickening	Fat Stranding
Sensitivity	0.84 [95%-CI: 0.62–0.94]	0.68 [0.56–0.77]	0.52 [0.44–0.59]	0.53 [0.47–0.60]	0.39 [0.32–0.48]
	Tau 2: 13.74	0.95	0.00	0.02	0.00
	Q: 17.12	74.90	7.44	7.83	2.54
	I 2: 76.60%	72.00%	19.30%	10.60%	0.00%
Specificity	0.83 [95%-CI: 0.75–0.89]	0.87 [0.80–0.92]	0.89 [0.82–0.94]	0.91 [0.82–0.96]	0.97 [0.94–0.98]
	Tau 2: 0.11	12.14	0.48	0.82	0.00
	Q: 7.20	142.75	23.15	31.68	1.7
	I 2: 44.40%	85.30%	74.10%	77.90%	0.00%
AUC (bivariate)	0.86	0.81	0.75	0.68	0.79

## Data Availability

Most data generated or analyzed during the study are included in the published paper. Additional data generated or analyzed during the study are available from the corresponding author by request.

## Appendix A. Search Strategy

Pubmed: (“Empyema, Pleural”[Mesh] OR (empyema* [tiab] OR pyothorax [tiab]) AND (pleura* [tiab] OR lung [tiab])) AND (“Tomography, X-Ray Computed”[Mesh] OR “computer assisted tomography”[tiab] OR “computed tomography”[tiab] OR “computed tomographic scan”[tiab] OR “computed tomography scan” [tiab] OR “computer tomography” [tiab] OR “computerized tomography” [tiab] OR “computerized tomography” [tiab]) AND (“Data Accuracy/statistics and numerical data”[Mesh] OR “Sensitivity and Specificity”[Mesh] OR “ROC Curve”[Mesh] OR “Area Under Curve”[Mesh] OR accuracy* [tiab] OR sens* [tiab] OR speci* [tiab] OR ROC [tiab] OR AUC [tiab]).

Embase: (‘Empyema, pleural’/exp OR (empyema*: ti, ab OR pyothorax: ti, ab) AND (pleura*: ti, ab OR lung: ti, ab)) AND (‘Tomography, X-Ray Computed’/exp OR “computer assisted tomography”: ti, ab OR “computed tomography”: ti, ab OR “computed tomographic scan”: ti, ab OR “computed tomography scan”: ti, ab OR “computer tomography”: ti, ab OR “computerized tomography”: ti, ab OR “computerized tomography”: ti, ab) AND (‘Data Accuracy/statistics and numerical data’/exp OR ‘Sensitivity and Specificity’/exp OR ‘ROC Curve’/exp OR ‘Area Under Curve’/exp OR accuracy*:ti,ab OR sens*:ti, ab OR speci*: ti, ab OR ROC: ti, ab OR AUC: ti,ab).

Web of Science: (“Empyema, pleural” OR (empyema* OR pyothorax) AND (pleura* OR lung)) AND (“Tomography, X-Ray Computed” OR “computer assisted tomography” OR “computed tomography” OR “computed tomographic scan” OR “computed tomography scan” OR “computer tomography” OR “computerized tomography” OR “computerized tomography”) AND (“Data Accuracy/statistics and numerical data” OR “Sensitivity and Specificity” OR “ROC Curve” OR “Area Under Curve” OR accuracy* OR sens* OR speci* OR ROC OR AUC).

## Appendix B. Extracted Data Items

The extracted data items were: (1) Expected outcome data were absolute numbers (number of true positive (TP), true negative (TN), false positive (FP), false negative (FN)) to calculate diagnostic accuracy measures, sensitivity, specificity, negative predictive value, positive predictive value, and AUC/ROC. (2) The various pleural findings were understood as prespecified thresholds for the diagnosis of empyema and were initially collected separately in the data collection since a threshold value for the diagnosis “empyema” from the combination of these findings has not yet been established. (3) A clear definition of the negative collective, as this is needed for comparison and pooling of the different identified studies (mainly: Parapneumonic effusions, benign effusions, and effusions in general). (4) A detailed description of computed tomography was included (vendor collimation, slice thickness, contrast, etc.). (5) Additional data items were: First author, published paper, study design, unit of assessment (per patient/ per effusion), prior testing, method of patient selection, number of participants, number of patients excluded (study overlap, insufficient test, no reference standard), number of empyemas (and prevalence), mean age, distribution of sex, definition of test positivity, thresholds of test positivity, number of readers and readers characteristics (e.g., years of professional experience), definition of the reference standard, the time interval between the reference standard and index test, country, year, follow-up, shortly conclusion, funding sources, and studied subgroups.

## Appendix C. Study Risk of Bias and Assessment of the Methodological Quality

A customized QUADAS-2 [15] was used, based on the four domains of “study selection”, “index test”, “reference standard”, and “flow and timing”. After the assessment, disagreements were resolved by consensus. During the pilot review, there was frequent disagreement on the reference standard because most studies had a clear definition of the reference standard, but the handling of indeterminate, or missing data, or blinding for index tests was unclear. In those studies, we rated the risk of bias as “unclear”, but concerns regarding applicability as “low” because of the accepted reference standard.

**Table A1:** Excluded studies.

First Author	Journal/Meeting	Publication Year	Reason for Exclusion
Schmitt [23]	Rofo	1981	No Diagnostic accuracy
Williford [24]	Radiol Clin North Am.	1983	No Diagnostic accuracy
Snow [25]	Chest	1990	No Diagnostic accuracy
Kohda [26]	Nihon Kyobu Shikkan Gakkai Zasshi	1994	No Diagnostic accuracy
Beigelman [27]	Rev Mal Respir.	1998	No Diagnostic accuracy
Kearney [28]	Clin Radiol.	2000	No Diagnostic accuracy
Ellis [29]	ER	2002	No Diagnostic accuracy
Smolikov [30]	Clin Radiol	2006	No Diagnostic accuracy
Lee [31]	J Comput Assit Tomogr.	2006	No Diagnostic accuracy
Heffner [32]	Chest	2010	No Diagnostic accuracy
Franklin [33]	BMJ	2011	No Diagnostic accuracy
Franklin [34]	AJRCCM	2012	No Diagnostic accuracy
Valdés [35]	Lung	2013	No Diagnostic accuracy
Yasnogorodsky [36]	Khirurgiia	2017	No Diagnostic accuracy
Carlucci [37]	Panminerva Med.	2019	No Diagnostic accuracy
Agrawal [38]	Indian Journal of Surgery	2020	No Diagnostic accuracy
Das [39]	Indian J Thorac Cardiovasc Surg	2021	No Diagnostic accuracy
Franklin [40]	Clinical Radiology	2021	No Diagnostic accuracy
Kendrick [41]	Pediatr Radiol.	2002	No Empyema
Ahmed [42]	Semin Interven Radiol	2012	Case report
Iudin [43]	Vestn Rentgenol Radiol	1997	No reference test
Liu [44]	Journal of Acute Medicine	2016	Empyemas as the negative collective

**Table A2:** Narrative summary of CT findings.

	CT Finding	TP	FN	FP	TN	Negative Collective	Sensitivity [95%-CI]	Specificity [95%-CI]	DOR [95%-CI]
Stark [49]	septated	10	47	3	9	A	17.5 [9.8; 29.4]	75.0 [46.8; 91.1]	0.0 [0.0; 2.8 ]
	smooth luminal margin	52	5	1	6	A	91.2 [81.1; 96.2]	85.7 [48.7; 97.4]	62.4 [6.2; 627]
Porcel [46]	microbubbles	13	10	24	103	A	56.5 [36.8; 74.4]	81.1 [73.4; 87.0]	5.6 [2.2; 14.2]
Metintas [50]	smooth margin	20	6	41	107	C	76.9 [57.9; 89.0]	72.3 [64.6; 78.9]	8.7 [3.3; 23.2]
	Moderate or large effusion	13	13	101	47	C	50.0 [32.4; 67.6]	31.9 [24.9; 39.7]	0.0 [0.0; 1.1]
Leung [51]	Lung base involvement	9	0	55	10	C	95.0 [65.5; 99.5]	15.9 [9; 26.6]	3.6 [0.0; 66.6]
Tsujimoto [47]	amount > 30 mm	26	10	14	33	B	72.2 [56.0; 84.2]	70.2 [56.0; 81.3]	6.1 [2.3; 16.0]
	gas pleural fluid	11	25	2	45	B	30.6 [18.0; 46.9]	95.7 [85.8; 98.8]	9.9 [2.0; 48.3]
	HU > 10	31	5	24	23	B	86.1 [71.3; 93.9]	48.9 [35.3; 62.8]	5.9 [2.0; 17.9]
	Septum	8	28	2	45	B	22.2 [11.7; 38.1]	95.7 [85.8; 98.8]	6.4 [1.3; 32.5]
Jimenez [48]	pleural gas	6	18	8	203	C	25.0 [12.0; 44.9]	96.2 [92.7; 98.1]	8.5 [2.6; 27.1]

Negative collective: A = parapneumonic, B = benign effusions, and C = effusions in general.

**Table A3:** Categorization of pleural findings, sensitivities, and specificities.

	Author	Neg. Collective	Threshold	TP	FN	FP	TN	Sensitivity [95%-CI]	Specificity [95%-CI]	DOR [95%-CI]
fat stranding	Jimenez [48]	C	visible	11	13	8	179	46 [28.3; 64.7]	95.5 [91.5; 97.6]	18 [6.3; 51.1]
		B	visible	11	13	2	84	46 [28.3; 64.7]	97.1 [91.2; 99.1]	28.8 [6.5; 126.9]
		A	visible	11	13	2	22	46 [28.3; 64.7]	90 [72.5; 96.8]	7.7 [1.7; 35.2]
	Waite [53]	B	visible	11	24	0	20	31.9 [19.1; 48.3]	97.6 [80.8; 99.8]	19.2 [1.1; 346.8]
		C	visible	12	23	0	50	34.7 [21.3; 51.1]	99 [91.3; 99.9]	53.7 [3.1; 946.3]
fat thickening	Jimenez [48]	B	visible	15	9	30	56	62 [42.6; 78.2]	64.9 [54.5; 74.1]	3 [1.2; 7.6]
		B	>2 mm	12	12	10	84	50 [31.8; 68.2]	88.9 [81.1; 93.8]	8 [2.9; 22.2]
		C	>2 mm	12	12	19	168	50 [31.8; 68.2]	89.6 [84.4; 93.2]	8.6 [3.5; 21.5]
		A	>2 mm	12	12	2	22	50 [31.8; 68.2]	90 [72.5; 96.8]	9 [2; 41.3]
	Waite [53]	B	visible	21	14	1	19	59.7 [43.5; 74]	92.9 [74.1; 98.3]	19.3 [3.2; 115.4]
		C	visible	21	14	4	26	59.7 [43.5; 74]	85.5 [69.2; 93.9]	8.7 [2.6; 29]
		C	3–4 mm	12	23	0	50	34.7 [21.3; 51.1]	99 [91.3; 99.9]	53.7 [3.1; 946.3]
loculation	Çullu [52]	C	visible	9	4	22	71	67.9 [42; 86]	76.1 [66.5; 83.6]	6.7 [2; 22.7]
		A	visible	9	4	9	38	67.9 [42; 86]	80.2 [66.9; 89]	8.6 [2.3; 32.3]
		B	visible	9	4	13	60	67.9 [42; 86]	81.8 [71.5; 88.9]	9.5 [2.7; 33.6]
	Jimenez [48]	B	visible	10	14	3	91	42 [25; 61.1]	96.3 [90.4; 98.6]	18.9 [5; 71.6]
		A	visible	10	14	2	22	42 [25; 61.1]	90 [72.5; 96.8]	6.5 [1.4; 30.1]
		C	visible	10	14	14	173	42 [25; 61.1]	92.3 [87.6; 95.3]	8.7 [3.3; 22.6]
	Stark [49]	A	visible	40	37	0	12	51.9 [41; 62.7]	96.2 [71.7; 99.6]	27 [1.5; 472.1]
pleural enhancement	Porcel [46]	A	split pleura	12	11	15	112	52.1 [33.2; 70.4]	87.9 [81.1; 92.5]	7.9 [3; 20.6]
	Stark [49]	A	split pleura	39	18	0	10	68.1 [55.3; 78.6]	95.5 [67.9; 99.5]	44.8 [2.5; 807]
	Tsujimoto [47]	B	split pleura	29	7	12	35	79.7 [64.3; 89.6]	74 [60.1; 84.3]	11.2 [4; 31.2]
	Waite [53]	C	visible	34	1	8	42	95.8 [83.8; 99]	83.3 [70.9; 91.1]	115 [19.1; 690.8]
		B	visible	24	1	8	20	94.2 [78.4; 98.7]	70.7 [52.5; 84]	39.4 [6.3; 246.1]
pleural thickening	Aquino [54]	C	2–4 mm	6	4	11	59	59.1 [31.6; 81.9]	83.8 [73.5; 90.6]	7.5 [1.9; 29.1]
		B	2–4 mm	6	4	8	52	59.1 [31.6; 81.9]	86.1 [75.2; 92.6]	8.9 [2.2; 36.3]
	Çullu [52]	A	visible	7	6	4	43	53.6 [29.6; 76]	90.6 [79.1; 96.1]	11.2 [2.7; 46.6]
		B	visible	7	6	5	68	53.6 [29.6; 76]	92.6 [84.3; 96.7]	14.4 [3.7; 56.2]
		C	visible	7	6	12	81	53.6 [29.6; 76]	86.7 [78.4; 92.1]	7.5 [2.2; 25.2]
	Jimenez [48]	B	costal	18	6	14	72	74 [54.5; 87.1]	83.3 [74.1; 89.7]	14.2 [4.9; 40.9]
		C	costal	18	6	57	130	74 [54.5; 87.1]	69.4 [62.5; 75.6]	6.5 [2.5; 16.6]
		A	costal	18	6	7	17	74 [54.5; 87.1]	70 [50.4; 84.3]	6.6 [1.9; 22.9]
		C	visceral	9	15	5	182	38 [21.8; 57.4]	97.1 [93.6; 98.7]	20.3 [6.3; 65.6]
		B	visceral	9	15	1	85	38 [21.8; 57.4]	98.3 [92.9; 99.6]	34.9 [5.7; 212.4]
		A	visceral	9	15	1	23	38 [21.8; 57.4]	94 [77.7; 98.6]	9.6 [1.5; 60.3]
	Leung [51]	B	smooth	8	1	6	20	85 [54.1; 96.5]	75.9 [57.3; 88.1]	17.9 [2.6; 125.3]
		C	visceral	9	0	11	15	95 [65.5; 99.5]	57.4 [39; 74]	25.6 [1.3; 486.5]
		B	unilateral	8	1	31	34	85 [54.1; 96.5]	52.3 [40.4; 63.9]	6.2 [1; 37.6]
		C	visceral	9	0	29	36	95 [65.5; 99.5]	55.3 [43.3; 66]	23.5 [1.3; 420.9]
	Metintas [50]	C	diffuse	15	11	59	109	57.4 [39; 74]	64.8 [57.3; 71.6]	2.5 [1.1; 5.7]
	Stark [49]	B	focal	11	15	5	25	42.6 [26; 61]	82.3 [65.5; 91.9]	3.4 [1; 11.4]
		C	focal	11	15	19	149	42.6 [26; 61]	88.5 [82.8; 92.4]	5.7 [2.3; 13.9]
		A	uniform	51	4	0	9	92 [81.9; 96.7]	95 [65.5; 99.5]	217.4 [10.8; 4378.8]
	Waite [53]	B	visible	30	5	0	20	84.7 [69.7; 93]	97.6 [80.8; 99.8]	227.4 [11.9; 4338.3]
		C	visible	30	5	8	42	84.7 [69.7; 93]	83.3 [70.9; 91.1]	27.7 [8.6; 89.3]
		B	3–4 mm	12	23	0	20	34.7 [21.3; 51.1]	97.6 [80.8; 99.8]	21.8 [1.2; 391.7]

Negative collective: A = parapneumonic, B = benign effusions, and C = effusions in general.

**Table A4:** DOR independent of negative collective.

DOR	Proportion [95%-CI]	Tau^2^	Q	AUC (Univariate)
enhancement	21.08 [7.91–56.20]	0.62	4.02	0.91
pleural thickening	10.11 [6.88–14.87]	0.29	20.38	0.82
loculation	9.40 [5.73–15.44]	0.00	2.15	0.79
fat thickening	7.99 [4.97–12.86]	0.05	6.91	0.80
fat stranding	17.88 [8.88–36.01]	0.00	2.15	0.81

**Table A5:** Diagnostic accuracy measures of pleural findings in benign effusions and effusions in general.

	CT Feature	Pooled Sensitivity [95%-CI]	Pooled Specificity [95%-CI]	AUC (Bivariate)	DOR [95%-CI]	Tau^2^	Cochrane Q	Heterogenity Chi^2^	AUC: Univariate
Benign Effusion	enhancement	0.89 [0.60–0.98]	0.73 [0.62–0.82]	0.76	20.1 [4.6–87.2]	0.5	1.00	2.28 *	0.93
	pleural thickening	0.64 [0.46–0.79]	0.86 [0.77–0,92]	0.85	13.5 [7.2–25.2]	0.2	8.00	10.10	0.84
	loculation	0.55 [0.26–0.82]	0.92 [0.64–0.99]	0.80	14.6 [5.6–38.4]	0.0	0.56	0.10 *	0.80
	fat thickening	0.59 [49.4–67.2]	0.87 [0.68–0.95]	0.61	8.7 [3.1–24.1]	0.5	3.03	7.06 *	0.87
	fat stranding	0.38 [0.26–0.53]	0.97 [0.92–0.99]	0.96	26.5 [7.1–99.0]	0.0	0.06	0.03 *	0.80
Effusion general	enhancement ^1^	0.97 [0.82–1.00]	0.84 [0.71–0.92]	0.97	7.9 [4.5–13.8]	NA	NA	NA	0.98
	pleural thickening	0.65 [0.51–0.78]	0.79 [0.66–0.88]	0.78	7.9 [4.6–13.8]	0.3	7.06	8.15	0.81
	loculation	0.56 [0.26–0.82]	0.86 [0.67–0.96]	0.78	8.2 [3.8–17.8]	0.0	0.06	0.34 *	0.75
	fat thickening	0.48 [0.32–0.64]	0.92 [0.79–0.97]	0.74	9.6 [4.8–19.6]	0.0	1.45	0.41	0.80
	fat stranding	0.40 [0.28–0.52]	0.96 [0.92–0.98]	0.77	20.4 [7.6–54.6]	0.0	0.49	0.06 *	0.80

Pleural findings marked with a ^1^ were only described in one study in the respective negative collective, which is why sensitivity, specificity, DOR, and AUC were not pooled and tau^2^, Cochrane Q, and Chi^2^ are not calculable (“NA”).* *p* < 0.05.

**Table A6:** Subgroup analysis.

		Sensitivity [95%-CI]	Tau^2^	I^2^	Specificity [95%-CI]	Tau^2^	I^2^
Random effect model		0.62 [0.55; 0.68]	0.7373	67.3%	0.90 [0.86; 0.93]	1.1359	82.5%
		**Sensitivity [95%-CI]**	***p***	**Q**	**Specificity [95%-CI]**	***p***	**Q**
Negative collective	All	0.63 [0.50; 0.74]	0.9234	0.16	0.88 [0.80; 0.93]	0.7485	0.58
	Benign	0.63 [0.52; 0.73]			0.91 [0.84; 0.95]		
	Parapneumonic	0.60 [0.48; 0.71]			0.90 [0.84; 0.94]		
Concerns regarding appicability	Yes	0.69 [0.58; 0.78]	0.1902	1.72	0.87 [0.80; 0.91]	0.3076	1.04
	No	0.60 [0.52; 0.68			0.90 [0.85; 0.93]		
Referencestandard for all patients	Yes	0.61 [0.54; 0.67]	0.2879	1.13	0.89 [0.85; 0.92]	0.9996	0.00
	No	0.75 [0.48; 0.90]			1.00 [0.00; 1.00]		
More than 1 reviewer	Yes	0.60 [0.52; 0.67]	0.5257	0.40	0.88 [0.83; 0.92]	0.2605	1.27
	No	0.65 [0.52; 0.76]			0.92 [0.86; 0.96]		
Slice thickness	10 mm	0.66 [0.52; 0.77]	0.634	1.71	0.94 [0.87; 0.98]	<0.001	84.39
	5 mm	0.62 [0.50; 0.72]			0.86 [0.80; 0.90]		
	3 mm	0.52 [0.32; 0.71]			0.88 [0.81; 0.93]		
Study after 2000	Yes	0.59 [0.50; 0.67]	0.4489	0.57	0.92 [0.87; 0.95]	0.0131	6.15
	No	0.63 [0.54; 0.71]			0.84 [0.78; 0.88]		
Pleural finding	pleural thickening	0.68 [0.56; 0.77]	0.0001	23.35	0.87 [0.80; 0.92]	<0.0001	24.68
	enhancement	0.84 [0.62; 0.94]			0.83 [0.75; 0.89]		
	fat stranding	0.39 [0.32; 0.48]			0.97 [0.94; 0.98]		
	fat thickening	0.53 [0.47; 0.60]			0.91 [0.82; 0.96]		
	loculation	0.52 [0.44; 0.59]			0.89 [0.82; 0.94]		
Empyema prevalence	<30%	0.59 [0.52; 0.65]	0.4491	0.57	0.87 [0.81; 0.91]	0.0387	4.27
	>30%	0.64 [0.52; 0.74]			0.94 [0.88; 0.97]		
High bias	Yes	0.60 [0.52; 0.66]	0.4270	0.63	0.88 [0.83; 0.92]	0.2291	1.45
	No	0.66 [0.51; 0.78]			0.93 [0.85; 0.97]		
